# Abortion in Cows1Presented at the Fourteenth Annual Meeting of the Iowa State Veterinary Medical Association, Des Moines, Iowa, February 11 and 12, 1902.

**Published:** 1902-05

**Authors:** Peter Malcolm

**Affiliations:** New Hampton, Iowa


### ABORTION IN COWS.[1]

By Peter Malcolm, V.S.,
NEW HAMPTON, IOWA.

This question is one of great importance to the veterinarian as well as to the breeder of cattle. Abortion, using the general meaning of the term, is the expulsion of the fœtus before it is viable.

The common causes of abortion in cows are external injuries, such as one animal butting another, squeezing through narrow places, slipping and falling, kicks from vicious attendants—in fact, any injury to the abdomen may produce it. Causes of a more obscure nature are internal, such as an abnormal or diseased condition of the uterus; inflammation of the bowels, kidneys, bladder, or lungs; indigestion in the acute or chronic form; evolution of gas in the intestines sufficient to cause irritation to the uterus or interfere with its circulation; diarrhœa, whether caused by irritant food or the reckless use of purgatives. The presence of a calculus in the kidney, ureter, bladder, or urethra may cause a sympathetic disorder of the uterus and expulsion of its contents. Irritant poisons that act on the urinary and generative organs, such as cantharides, savin, tansy, ergot, smut, and various fungi that are found in decomposing vegetable matter. Another cause, and one of great importance, is bad ventilation, or any like condition which interferes with the normal oxidation of the blood. The importance of keeping pregnant animals in well-ventilated stables can be seen at a glance, when you take into consideration the condition of their blood, which contains an excess of water and a smaller proportion of albumin and red corpuscles. This condition aggravated by bad ventilation, decomposed animal and vegetable matter, poor food and stagnant water is almost sure to result in abortion.

The dam with all her diseases and the accidents that may be forced upon her is not the sole cause of abortion. To the sire a great deal of this trouble is due, and this should not be lost sight of, as he plays a prominent part in the transmission of disease. In the first place, it is not reasonable to suppose that a sire that is overworked can produce strong and vigorous spermatozoa. When this weakened spermatozoön comes into contact with the ovum, the chemical constituent will be of a debilitated character, which

[1] Presented at the Fourteenth Annual Meeting of the Iowa State Veterinary Medical Association, Des Moines, Iowa, February 11 and 12, 1902.

will, if it develops, ultimately cause disease of the fœtus or its envelops. Furthermore, this overworked sire is in a condition, on account of the weakened state of his generative organs, which furnish a favorable field for the development of vigorous microbes, which, when the act of copulation is performed, are carried into the vagina. Together with the spermatozoa these germs enter the uterus, and there develop, causing disease of the fœtus or its envelope, which may bring about abortion, or, if not, will produce disease of the offspring.

Another cause, and one of great importance, is infection. In some instances its origin is obscure, but the majority of outbreaks can be traced to neglected cases of simple or accidental abortion. In this form of abortion there is no longer a doubt as to the pathogenic agent, as science has proven beyond a doubt that it is due to a microörganism. Such conditions exist, and we are called upon to treat them. To do this successfully, it is necessary to understand the character and pathological action of this organism. It is a pathogenic microbe, developed in decomposing animal or vegetable matter. It enters the system by way of the respiratory or digestive tract, the vagina, or any abrasion of the skin. Gaining access to the blood, it causes putrefactive fermentation, which produces an irritation to the sympathetic system and death to the fœtus.

The treatment of this disease, or, more properly speaking, this deuteropathy, requires tact and energy, as the condition and circumstances that favor its progress are numerous and of an obscure nature. Overlooking a seemingly trivial condition may lead to serious consequences. An essential point to be considered in the preventive treatment is to see that the sire and dam are in a healthy condition before mating them. The sire should be kept away and not allowed to run with the cows, nor should he be allowed for at least three months or more to have intercourse with a cow that has aborted, and then should be allowed only one service. On no day should he serve more than three cows. The cow that has aborted should not be bred until after the period at which she would have given birth naturally, for in the majority of cases, if an aborting cow becomes impregnated she will abort when that period is reached.

In the treatment of a herd for abortion, do not wait to see if it is going to take on the epizoötic form, for delay is dangerous. One neglected case, no matter what the cause is, may cause abortion to every cow in the herd. Therefore, it is very essential to remove the cows that have aborted, thoroughly disinfect them, burn the placenta, destroy the fœtus and all other débris that may have become contaminated with the fluids, and disinfect the stable. For disinfection I would advise carbolic acid, as my experience has taught me to believe that carbolic acid is not only a specific in the destruction of this particular microbe, but that it arrests the fermentative changes that favor its development. In using carbolic acid in cases of this nature, two things should be noted: first, that the inhalation of the fumes is necessary, inasmuch as they arrest and destroy germs that may have gained access to the air-passages; second, that if used too freely it may cause an irritation to the respiratory organs sufficient to produce inflammation of the lungs. A safe formula, and one of sufficient strength, is one ounce of carbolic acid, one-half ounce of glycerin, and sixteen ounces of warm water, applied once a day for four or five days to the floor of the stable and to the rumps and tails of the cows. Internally, given in drinking water once a day for three or four days, about four drachms of hyposulphite of soda. Again, allow me to impress upon your mind that the microbes must gain access to the blood before they can do any harm. Also, that the injecting of the vagina is useless and that the irritation thus produced will cause abortion; and that the success in mastering this disease depends on the sanitary conditions.

### Discussion.

Dr. Scott asked Dr. Malcolm if he has by his method ever been able to arrest the disease when once well established in a herd. Dr. Malcolm said that in one herd of twenty cows nineteen aborted in one year. He treated the herd, and the owner got a new bull. The next year only a few aborted. In another herd of fifteen cows seven aborted in one year. He treated them, and, although three years have elapsed since, there have been no more abortions. He always has the stables disinfected when the cows are put in in the fall.

---

The many friends of Dr. William H. Lowe, of Paterson, N. J., treasurer of the A. V. M. A., will regret to learn of his great loss by the fire holocaust in February that destroyed almost the entire business section of Paterson. Dr. Lowe's loss involved his hospital and all its contents, save his ambulance, which was afterward destroyed by the flood that succeeded the great fire.

